# The Molecular Mechanisms Underlying Mitochondria-Associated Endoplasmic Reticulum Membrane-Induced Insulin Resistance

**DOI:** 10.3389/fendo.2020.592129

**Published:** 2020-11-23

**Authors:** Han Cheng, Xiaokun Gang, Guangyu He, Yujia Liu, Yingxuan Wang, Xue Zhao, Guixia Wang

**Affiliations:** Department of Endocrinology and Metabolism, the First Hospital of Jilin University, Changchun, China

**Keywords:** mitochondria-associated endoplasmic reticulum membrane, mitochondria, endoplasmic reticulum, insulin resistance, type 2 diabetes, endoplasmic reticulum stress

## Abstract

Mitochondria and the endoplasmic reticulum (ER) are connected at multiple sites *via* what are known as mitochondria-associated ER membranes (MAMs). These associations are known to play an important role in maintaining cellular homeostasis. Impaired MAM signaling has wide-ranging effects in many diseases, such as obesity, diabetes, and neurodegenerative disorders. Accumulating evidence has suggested that MAMs influence insulin signaling through different pathways, including those associated with Ca^2+^ signaling, lipid metabolism, mitochondrial function, ER stress responses, and inflammation. Altered MAM signaling is a common feature of insulin resistance in different tissues, including the liver, muscle, and even the brain. In the liver, MAMs are key glucose-sensing regulators and have been proposed to be a hub for insulin signaling. Impaired MAM integrity has been reported to disrupt hepatic responses to changes in glucose availability during nutritional transition and to induce hepatic insulin resistance. Meanwhile, these effects can be rescued by the reinforcement of MAM interactions. In contrast, several studies have proposed that enhanced ER-mitochondria connections are detrimental to hepatic insulin signaling and can lead to mitochondrial dysfunction. Thus, given these contradictory results, the role played by the MAM in the regulation of hepatic insulin signaling remains elusive. Similarly, in skeletal muscle, enhanced MAM formation may be beneficial in the early stage of diabetes, whereas continuous MAM enhancement aggravates insulin resistance. Furthermore, recent studies have suggested that ER stress may be the primary pathway through which MAMs induce brain insulin resistance, especially in the hypothalamus. This review will discuss the possible mechanisms underlying MAM-associated insulin resistance as well as the therapeutic potential of targeting the MAM in the treatment of type 2 diabetes.

## Introduction

Type 2 diabetes mellitus (T2DM) is a metabolic disease characterized by hyperglycemia. High levels of circulating glucose can result from defective insulin secretion, insulin resistance, or both. In the early stages of T2DM, most patients exhibit insulin resistance. Beta-cells increase insulin output as a compensatory response to maintain normal glucose tolerance. With the progression of the disease, dysfunctional β-cells do not release sufficient insulin to compensate for peripheral insulin resistance, leading to the development of overt T2DM (1). The incidence of T2DM has greatly increased worldwide in recent years, and no cure is yet available for this disorder (2). Therefore, it is essential to better understand the mechanisms underlying this pathophysiology so that novel therapeutic approaches can be developed.

Mitochondria and the endoplasmic reticulum (ER) are two essential organelles that share structural and functional communication to maintain cellular homeostasis (3). The regions of close contact between these two organelles are known as mitochondria-associated ER membranes (MAMs). MAMs play an important role in various cellular processes ranging from cell signaling and metabolite transport to cell death and survival (4). These cellular processes are also partially involved in the insulin signaling pathway, and several proteins in this pathway, such as protein kinase B (PKB/AKT), mammalian target of rapamycin complex 2 (mTORC2) (5), and phosphatase and tensin homolog (PTEN) (6), can localize to MAMs and interact with MAM-resident proteins. This suggests that the MAM might serve as a key regulator of insulin signaling. Moreover, accumulating evidence has revealed that dysregulated communication between the ER and mitochondria is associated with diverse pathophysiological conditions, including metabolic diseases such as T2DM, obesity, and neurodegenerative diseases (7). However, the mechanisms underlying MAM-induced insulin resistance remain elusive and the results of studies to date are contradictory. In this review, we mainly discuss how the MAM regulates insulin signaling and the possible pathway through which altered MAM signaling leads to insulin resistance in different tissues. Finally, we hypothesize that the MAM might be a promising therapeutic target for the treatment of T2DM.

## The Structural Composition of the Mam

To date, more than a thousand proteins have been identified in isolated MAMs (8–10). These proteins have been classified into the following three groups: 1) MAM-resident proteins that only localize to the MAM; 2) MAM-enriched proteins that can also be found in other regions of the cell; and 3) MAM-associated proteins that are transiently found in the MAM in a condition-dependent manner (9). Owing to the highly dynamic nature of the MAM, the detailed characterization of its components has remained elusive.

Some of the candidate proteins are important tethers that bridge the gap between the ER and mitochondria, and nearly all these linker proteins also have other functions. In mammalian cells, these tethers can be singular, bipartite, or multipartite. Mitochondria-localized ATPase family AAA domain-containing protein 3A (ATAD3A) is known to interact with the ER membrane through its N-terminus, and is the only single-protein linker between the ER and mitochondria. Besides its role as a physical linker, ATAD3A can also regulate the import of phosphatidylserine (PS) into mitochondria from the ER and modulate mitochondrial morphology (11). ER-localized mitofusin 2 (MFN2) homodimers or heterodimers formed between MFN2 and MFN1 on the mitochondrion were the first proposed bipartite tethers (12). However, their structural role in the MAM remains unclear. Some studies have suggested that MFN2 promotes ER–mitochondria contacts (12–14), whereas others have reported that it acts as a tethering antagonist (15, 16). Meanwhile, its interaction with the familial Alzheimer’s disease-related protein presenilin-2, initially proposed to negatively affect MAM functions (17), was also challenged by other studies. These studies demonstrated that presenilin 2 can block the inhibitory effects of MFN2 and positively modulate ER–mitochondria coupling (18). Tethering interactions that may compensate for MFN2 loss under specific conditions have also been identified. One of these tethers is formed by ER-localized vesicle-associated membrane protein-associated protein B and C (VAPB) and the mitochondrial protein tyrosine phosphatase-interacting protein 51 (PTPIP51). The loss of either impairs Ca^2+^ transfer from the ER to mitochondria (19) and stimulates autophagy. However, autophagosome formation may involve factors other than the loss of VAPB or PTPIP51 because overexpressing an artificial ER–mitochondrion tether rescues this autophagy-related effect. Thus, it might be the loosened ER–mitochondria contacts and the ensuing dysregulated Ca^2+^ transport that mediate autophagy (20). An interaction formed by ER-localized B-cell receptor-associated protein 31 (Bap31) and the mitochondrial fission protein fission 1 homolog (Fis1) has also been identified. The Fis1/Bap31 platform is required for the activation of procaspase-8, resulting in the cleavage of Bap31 into the proapoptotic p20Bap31 fragment (21) and the rapid transfer of Ca^2+^ from the ER to the mitochondria *via* the inositol 1,4,5-triphosphate receptor (IP3R) complex (22). In mitochondria, this Ca^2+^ influx promotes cristae remodeling followed by the release of cytochrome c, which eventually leads to apoptosis (23). The tripartite IP3R complex, comprising the ER-resident IP3R, the mitochondria-localized voltage-dependent anion channel 1 (VDAC1), and the cytosolic chaperone glucose-regulated protein 75 (GRP75), forms a tether between the ER and mitochondria and is involved in Ca^2+^ transfer between the two organelles. However, ER–mitochondria associations are unaffected in IP3R-knockout cells (8), indicating that the IP3R complex is more likely to provide a platform for Ca^2+^ transfer rather than being a structural tether. Recently, etoposide-induced protein 2.4 (EI24), which regulates autophagic flux, was reported to interact with VDAC1 and possibly form a quaternary complex with IP3R, GRP75, and VDAC1 (24). However, after DNA damage, EI24 was found to interact with VDAC2 and not VDAC1 (25), suggesting that MAM composition might vary under different conditions.

**Figure 1:**
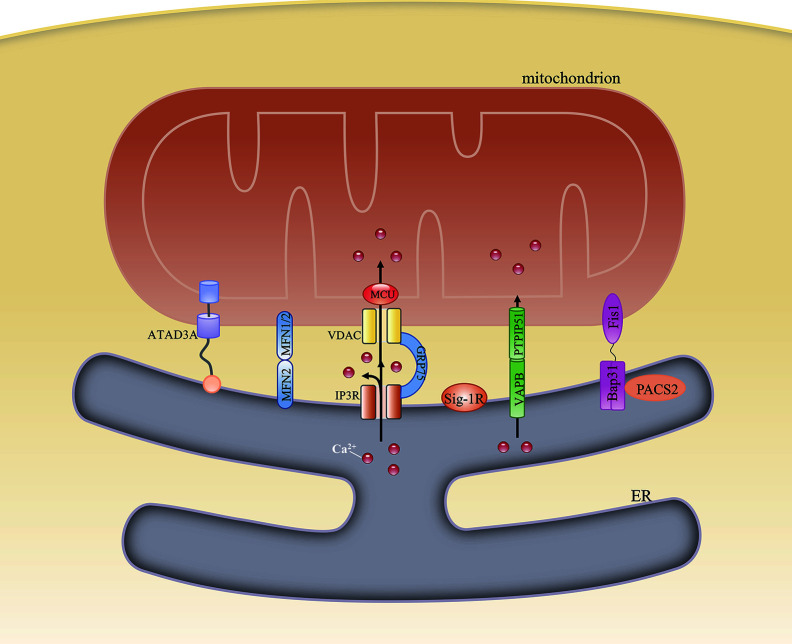
Structural composition of the mitochondria-associated endoplasmic reticulum (ER) membrane (MAM). The structure of the MAM is maintained by interactions among various MAM-resident proteins. ATAD3A is the only single-protein linker at the MAM. Bipartite complexes are formed between MFN2 and MFN1/2, VAPB and PTPIP51, and Bap31 and Fis1. The tripartite tether comprises IP3R, GRP75, and VDAC. Besides their role in maintaining ER–mitochondria contacts, these proteins can also regulate the transfer of some metabolites between the two organelles.

In addition to the above-mentioned proteins, others have also been found to be involved in MAM formation, including phosphofurin acidic cluster sorting protein 2 (PACS2) (26), PDZ domain-containing protein 8 (PDZD8) (27), and sigma 1 receptor (Sig-1R) (28), among others (29) (**Figure 1**). The function of each of these proteins under specific conditions is discussed below.

## The Mam-Mediated Regulation of Cellular Homeostasis and Its Relationship With Insulin Resistance

MAMs are now known to be specialized lipid raft-like regions. These lipid microdomains are a dynamic assemblage of sphingolipids and cholesterol that can move within the fluid lipid bilayer and function as platforms for the attachment of proteins during signal transduction (30). These proteins can be enzymes, transporters, kinases, ion channels, or phosphatases (9), indicating that MAMs participate in various cellular processes, such as calcium homeostasis, lipid metabolism, mitochondrial physiology, ER stress, and inflammation. Almost all of the above processes have been reported to interact with insulin signaling, and their disruption is closely associated with the loss of both insulin action and secretion in T2DM.

### Calcium Homeostasis

MAMs are important hubs for Ca^2+^ signaling. When cells are exposed to stimuli, such as the firing of action potentials in neurons or following cell injury, a large amount of Ca^2+^ is released from the ER through IP3Rs and ryanodine receptors (RyRs). Given their minuscule size, even a small Ca^2+^ flux into the MAM microdomain would be greatly amplified. These high-Ca^2+^ microdomains are required for the induction of mitochondrial Ca^2+^ uptake through the low-affinity mitochondrial Ca^2+^ uniporter (MCU) (31). Moreover, Ca^2+^ transfer between the ER and mitochondria is dependent on the cooperation of ER–mitochondria tethering proteins. These proteins can keep the ER–mitochondria contact at a proper distance. Deficiencies in tethering proteins, such as PDZD8, were recently reported to significantly reduce mitochondrial Ca^2+^ import and impair cytosolic Ca^2+^ dynamics (30). On the one hand, mitochondrial Ca^2+^ uptake protects cells from significant Ca^2+^ fluctuations and maintains Ca^2+^ signaling. On the other hand, Ca^2+^ is required for mitochondrial ATP production as Ca^2+^ regulates the activity of the tricarboxylic acid cycle, as well as that of several mitochondrial enzymes involved in ATP synthesis (32). If ER–mitochondria tethering is enhanced, excessive Ca^2+^ transfer from the ER to mitochondria will lead to mitochondrial Ca^2+^ overload and oxidative stress. If the ER–mitochondria contact is weakened, excessive Ca^2+^ release to the cytoplasm will lead to a cytosolic Ca^2+^ wave. Additionally, inadequate mitochondrial Ca^2+^ influx leads to impaired mitochondrial respiration and decreased ATP production.

Ca^2+^ signaling influences various aspects of glucose metabolism, including glucogenesis and glucose utilization. First, during fasting, high levels of glucagon can indirectly lead to the phosphorylation and activation of IP3R, resulting in increased cytosolic Ca^2+^ concentrations. Higher cytosolic Ca^2+^ concentrations can induce gluconeogenesis by inducing the calcineurin-mediated dephosphorylation of CREB coactivator (CRTC2), which can promote the expression of peroxisome proliferator-activated receptor gamma coactivator 1-alpha (PGC1α) and the subsequent modulation of gluconeogenic gene expression (33). Excessive gluconeogenesis is an important contributor to hyperglycemia in insulin resistance. Furthermore, IP3R activity is increased in diabetes. IP3R can indirectly upregulate the expression of gluconeogenic genes and promote gluconeogenesis, leading to elevated circulating glucose levels (34). Second, Ca^2+^ signaling affects glucose utilization by regulating the insulin signaling pathway. The G protein/IP3/IP3R pathway is involved in glucose transporter 4 (GLUT4)-plasma membrane fusion, while pharmaceutically induced GLUT4-plasma membrane fusion and glucose uptake are inhibited by treatment with a Ca^2+^ chelator, suggesting that these processes are Ca^2+^-dependent (35).

**Figure 2:**
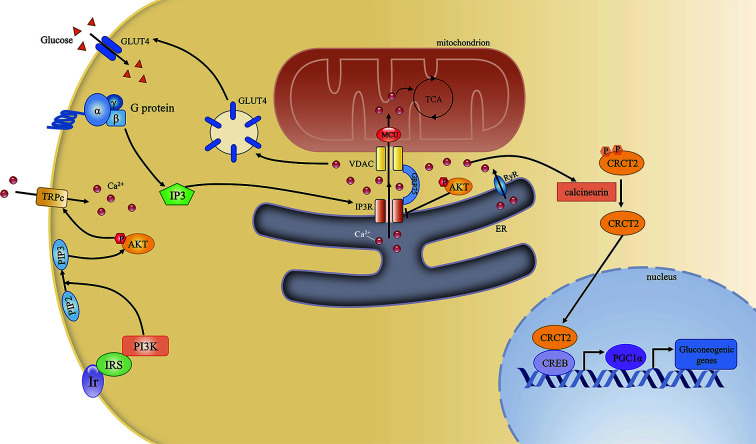
The mitochondria-associated endoplasmic reticulum (ER) membrane (MAM) regulates cellular calcium homeostasis and insulin signaling. The complex comprising PI3R, GRP75, and VDAC is the key mediator of Ca^2+^ transport from the ER to mitochondria. Ca^2+^ is an important second messenger that regulates various cellular processes. First, Ca^2+^ is required for mitochondrial ATP production. Second, during fasting, elevated Ca^2+^ release from the ER through IP3R and RyR can induce gluconeogenic gene expression *via* the CRCT2/PGC1α pathway. Third, the G protein/IP3/IP3R pathway can regulate GLUT4–plasma membrane fusion by modulating Ca^2+^ levels. Conversely, insulin signaling can also promote cellular Ca^2+^ influx by activating transient receptor potential channels (TRPCs).

Conversely, insulin signaling may also affect Ca^2+^ homeostasis. Activated AKT induces the opening of transient receptor potential channels (TRPCs) and the subsequent Ca^2+^ influx. Elevated intracellular Ca^2+^ levels result in the opening of Ca^2+^ channels in the ER and facilitate mitochondrial Ca^2+^ uptake (36). Furthermore, phosphorylated AKT can be recruited to the MAM interface in response to insulin signaling, while an *in situ* proximity ligation assay showed that both AKT and phosphorylated AKT localized in close proximity to IP3R1 (37). AKT can directly phosphorylate and inhibit the function of IP3R, resulting in decreased ER Ca^2+^ release and attenuated cytosolic Ca^2+^ signaling (38) (**Figure 2**).

A meta-analysis identified an association between a single-nucleotide polymorphism (SNP) in the IP3R2 locus and alterations in the waist-to-hip ratio adjusted for body mass index (39), suggesting that Ca^2+^ flux through IP3R has a role in human metabolic disease. Combined, the above results indicate that MAM-resident IP3R-regulated Ca^2+^ signaling is an important modulator of glucose metabolism, and that impaired Ca^2+^ flux is associated with obesity and/or possibly also the development of T2DM.

### Lipid Metabolism

The MAM is also associated with the activity of diverse enzymes that regulate lipid metabolism. The best-studied of these are involved in the transfer of phospholipids between the ER and mitochondria. First, PS is synthesized at the MAM by PS synthase 1/2 (PSS1/2). PS is then transferred to the near apposed mitochondrion and decarboxylated by PS decarboxylase (PSD), yielding phosphatidylethanolamine (PE) in the inner mitochondrial membrane. Finally, PE returns to the ER and is converted to phosphatidylcholine (PC) by phosphatidylethanolamine N-methyltransferase 2 (PEMT2) (40). The PC/PE ratio plays a key role in regulating cell membrane integrity, and alterations in this ratio contribute to the progression of steatosis into steatohepatitis (41). In addition, an increased PC/PE ratio can inhibit ER Ca^2+^ transport and induce ER stress and hyperglycemia in models of obesity (42). That PS synthase localizes exclusively to the MAM and the transfer of PS from the ER to mitochondria is the rate-limiting step in PE synthesis highlights the importance of the MAM in maintaining the PC/PE ratio (43). Cells lacking multiple MAM components exhibit reduced PS transfer from the ER to mitochondria (44). Furthermore, MFN2 was recently reported to mediate PS transfer to mitochondria. MFN2 binds PS and can specifically extract PS into membrane domains, favoring its transfer to mitochondria and mitochondrial PE synthesis (45).

The MAM is also involved in cholesterol transport and metabolism. Recently, caveolin 1 was identified at the MAM interface, where it was shown to regulate ER–mitochondria cholesterol transfer (46). Moreover, MAM-localized VDAC2 and translocator proteins are key mediators of cholesterol transport from the cytosol to mitochondria (47). Acyl-coenzyme A (CoA):cholesterol acyltransferase located in the MAM catalyzes the conversion of free cholesterol to cholesteryl esters, which maintains the balance between membrane-bound and cytoplasm-stored cholesterol in the resting state (48). Cholesterol is the main lipid component of cellular membranes and also plays an important role in signaling. In the brain, cholesterol can be oxidized into oxysterols, which can impair neuronal glucose uptake through the modulation of GLUT4 activity (49). Cholesterol depletion in hypothalamic neurons contributes to insulin resistance and enhanced apoptosis (50). In turn, insulin signaling also serves as a key regulator of cellular cholesterol metabolism. PI3K/AKT/mTOR activation can enhance cholesterol levels (51).

The conversion of diacylglycerols (DAGs) to triglycerides (TAGs) can be catalyzed by MAM-resident diacylglycerol acyltransferase 2 (DGAT2) (52). Impaired MAM integrity has been reported to lead to mitochondrial dysfunction. Dysfunctional mitochondria showed decreased fatty acid oxidation, leading to increased fatty acyl CoA and DAG levels. The increased concentrations of these molecules induced Ser/Thr kinase activity, thereby enhancing the serine phosphorylation of insulin receptor substrate 1 (IRS-1) and blocking the tyrosine phosphorylation of IRS-1 by the insulin receptor. As a consequence, insulin-induced glucose uptake was suppressed (53). A different study reported that DAGs activated protein kinase C-theta (PKCθ), which promoted IRS-1 Ser1101 phosphorylation in muscle, suppressed IRS-1 tyrosine phosphorylation, and impaired insulin signaling. Mice overexpressing DGAT2 in the liver manifested severe insulin resistance, which was attributed to increased DAG-induced PKCϵ activation. Activated PKCϵ decreased the insulin-stimulated tyrosine phosphorylation of IRS-2 and increased the pAKT/AKT ratio (54). A reduction in the expression of DGAT2 can improve hepatic insulin sensitivity (55).

**Figure 3:**
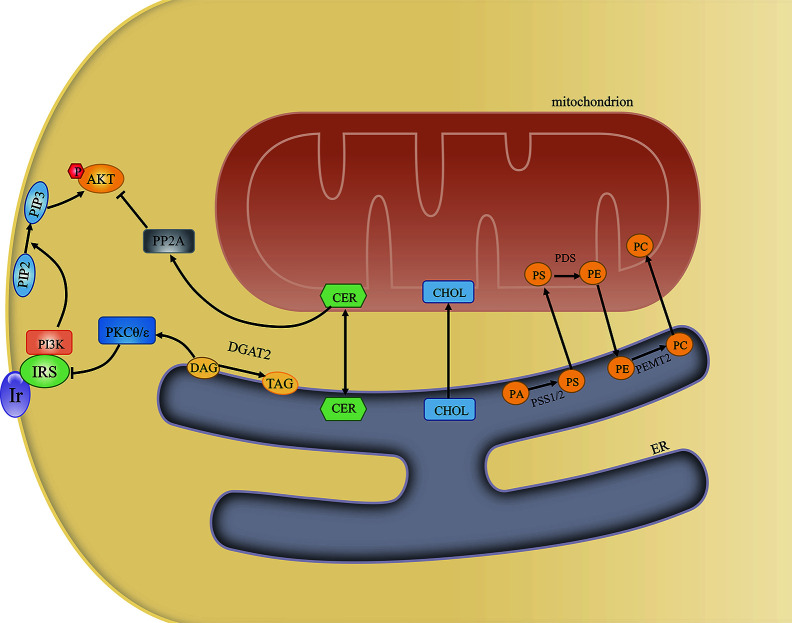
The mitochondria-associated endoplasmic reticulum (ER) membrane (MAM) regulates cellular lipid metabolism and insulin signaling. The MAM is an important site for lipid metabolism, including phosphatidylcholine (PC) and ceramide synthesis, cholesterol transport, and diacylglycerol (DAGs)-to-triglyceride (TAG) conversion. Excessive ceramide and DAG generation can inhibit insulin signaling by activating PKCθ/ϵ and PP2A, respectively.

The enzymes sphingomyelin phosphodiesterase and ceramide synthase have also been identified in MAMs, indicating that this contact site can generate a certain amount of ceramide (56, 57). Under normal conditions, ceramides can be transferred to mitochondria and converted to sphingosine-1-phosphate and hexadecenal (58). When ER–mitochondria contacts are disrupted, ceramides are not transferred to mitochondria, leading to increased cytoplasmic ceramide levels. Elevated ceramide concentrations are usually associated with insulin resistance. Ceramides activate protein phosphatase 2 alpha (PP2A), which dephosphorylates AKT and subsequently suppresses its activation (59). Ceramides also stimulate PKCζ, preventing the association of AKT with the membrane and thereby inhibiting AKT activity and insulin signaling (60) (**Figure 3**). In obese rats, attenuating increased ceramide levels can ameliorate insulin sensitivity in the hypothalamus and prevent central insulin resistance (61). Equally, impaired insulin signaling may further lead to elevated ceramide levels and ceramide-induced activation of atypical PKC, which aggravates insulin resistance (62). In summary, the MAM may help maintain proper insulin signaling by sustaining lipid homeostasis.

### Mitochondrial Physiology

In addition to its role in regulating mitochondrial Ca^2+^ transport, MAM activity also affects mitochondrial physiology, including mitochondrial bioenergetics, dynamics, and mitophagy.

The MAM can control mitochondrial oxidative metabolism mainly by regulating Ca^2+^ transfer. Inositol-requiring protein 1 alpha (IRE1α) is a key mediator of ER stress. It also localizes to the MAM where it physically interacts with IP3R and regulates mitochondrial Ca^2+^ uptake. IRE1α deficiency can impair the tricarboxylic acid cycle, whereas overexpression of IP3R can rescue this effect and increase mitochondrial ATP production (63). Furthermore, the mitochondria-resident protein translocase of outer mitochondrial membrane 70 (TOMM70) can also sustain mitochondrial respiration by associating with IP3R and promoting Ca^2+^ shuttling between the ER and mitochondria (64). Moreover, another ER membrane-associated protein, Bap31, was reported to form an ER–mitochondria bridging complex with mitochondrial-resident proteins such as TOMM40. These complexes are important for the activation of the mitochondrial respiratory chain through the regulation of complex I activity (65). In the liver, a high glucose concentration can disrupt MAM integrity and reduce mitochondrial respiration through the PP2A pathway (66). These results indicate that MAM-mediated mitochondrial bioenergetics not only regulate insulin secretion but are in turn also regulated by insulin signaling.

Mitochondrial dynamics include mitochondrial fission, fusion, and motility. During fission, dynamin-related protein 1 (DRP1) is recruited to the outer mitochondrial membrane, forming a helix around mitochondria that constricts and divides the mitochondrion into two parts (67). However, mitochondrial constriction can occur at or near sites of contact with the ER even in the absence of DRP1, suggesting that ER tubules may precede mitochondrial fission and define the position of mitochondrial division sites (68). Other proteins that regulate mitochondrial fission, such as inverted formin 2 (INF2) (69), syntaxin 17 (STX17), and Rab32 (70), are subsequently also detected at the MAM. Decreased MAM formation was proposed to cause mitochondrial elongation and dysfunction, effects that are associated with decreased mitochondrial fission. Mechanistically, decreased MAM formation can lower both mitochondrial and cytosolic Ca^2+^ concentrations. Reduced intracellular Ca^2+^ levels can inhibit the binding of cAMP response element binding protein (CREB) to the *Fis1* promoter, thereby suppressing *Fis1* expression and mitochondrial fission (71).

Mitochondrial fusion is mainly mediated by MFN proteins on the outer mitochondrial membrane and optic atrophy 1 (OPA1) on the inner mitochondrial membrane. MFN2 forms either homodimers or heterodimers with MFN1 to promote mitochondrial tethering and, subsequently, mitochondrial fusion. Mitochondrial fusion allows for the exchange of contents between mitochondria, as well as the rescue of defective mitochondria for the recovery of essential components (72). A recent study proposed that the fission and fusion machineries assemble at the same ER–mitochondria contact site to modulate mitochondrial morphology in response to external insults and metabolic cues, such as the nutrient status (73), while MFNs also accumulate at the MAMs where fusion occurs. However, it remains unclear how the positions of mitochondrial fusion sites are determined. The specialized lipid environment of the MAM is thought to promote membrane curvature and favor both membrane fission and fusion. Furthermore, the high-Ca^2+^ MAM microdomain is an important stimulator of fission and fusion (74). Moreover, it is still controversial whether disrupted ER–mitochondria contacts lead to mitochondrial elongation or mitochondrial fragmentation. Wu et al. proposed that decreased MAM formation lowered both mitochondrial and cytosolic Ca^2+^ levels. The reduced intracellular Ca^2+^ concentration inhibited *Fis1* expression and mitochondrial fission, resulting in mitochondrial elongation (71). In contrast, Puri et al. found that impaired ER–mitochondria contact increased cytosolic Ca^2+^ levels, which indirectly activated DRP1 through the activation of calcineurin phosphatase and led to mitochondrial fragmentation (75). Although the reason for this contradiction remains unknown, we speculate that it may be associated with the degree of MAM integrity impairment. Consequently, we suggest that future experiments should focus on measuring the distance between the ER and mitochondria to better understand the relationship between changes in mitochondrial morphology and ER–mitochondria contact.

Mitochondrial motility is defined as mitochondrial movement along microtubules throughout the cell. The connection between mitochondria and microtubules is mainly mediated by mitochondrial Rho GTPase 1 (MIRO1) and MIRO2 (76). There is evidence to support that the role of the MAM in mitochondrial motility is tightly associated with MIRO1/2. First, the yeast ortholog of MIRO1 is reported to localize to sites of ER–mitochondrial contact (77), while the loss of MIRO1/2 alters mitochondria–ER communication (78). Second, the ER stays attached to the mitochondrion as it is being transported throughout the cell. Third, MAM-localized MFN2, which interacts with MIRO1/2, is necessary for axonal mitochondrial transport (79). Fourth, the docking of a mitochondrion at a specific position relies on the binding of Ca^2+^ to the EF-hand motifs in MIRO1/2, which disconnects the organelle from the microtubule (80). However, such binding needs a high Ca^2+^ concentration given the low affinity of MIRO1/2 for Ca^2+^. Therefore, the MAM may serve as a Ca^2+^ source that determines the sites of mitochondria redistribution (81). Increased ER–mitochondria contact and Ca^2+^ transfer may lead to defects in axonal mitochondrial transport (82).

**Figure 4:**
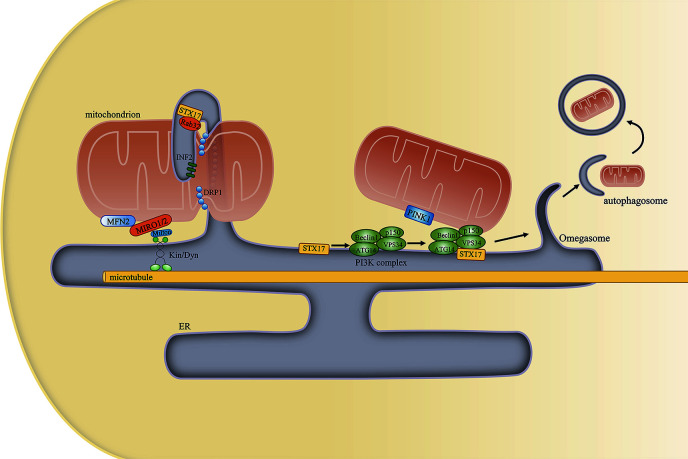
The mitochondria-associated endoplasmic reticulum (ER) membrane (MAM) regulates mitochondrial physiology. (Left) Mitochondrial constriction occurs near the sites of contacts with the ER, and the ER can define the position of mitochondrial fission. Other proteins that are localized at the MAM, such as STX17, Rab32, and INF2, also participate in this process. Furthermore, the ER remains attached to mitochondria and moves with it along microtubules in the cell. (Right) MAM-resident STX17 binds to ATG14 and recruits the class III PI3K complex to the MAM, which facilitates autophagosome formation.

Mitophagy represents a selective autophagic process for the elimination of damaged mitochondria. Mitophagy can be classified into six steps: induction of autophagy, nucleation of the isolation membrane (also known as the phagophore), expansion of the isolation membrane, formation of the autophagosome, fusion of the autophagosome with a lysosome to form an autolysosome, and degradation and recycling. PTEN-induced putative kinase 1 (PINK1), a key regulator of mitophagy, and Beclin1 both relocalize to the MAM, enhancing ER–mitochondria contact and promoting autophagosome formation following autophagy induction (83). Beclin1 is also a key component of the class III PI3K complex that produces PI3P, a contributor to autophagosome formation (84). Another mitophagy-associated protein, FUN14 domain-containing protein 1 (FUNDC1), was also shown to accumulate at the ER–mitochondria interface during mitophagy by binding to ER-resident IP3R2 (71, 85). A different study revealed that MAM-resident STX17 can bind the autophagosome marker ATG14 and recruit it to the MAM until the completion of autophagosome formation. This supports that the autophagosome forms at MAMs (86) (**Figure 4**).

Mitochondrial fission, fusion, and mitophagy together constitute the mitochondrial quality control system that serves to maintain mitochondrial homeostasis. The mitochondrion plays an important role in regulating insulin signaling (36). Consequently, the impairment of the mitochondrial quality control system, which leads to mitochondrial dysfunction, is closely associated with T2DM. In the hippocampus of *db/db* mice, activated glycogen synthase kinase 3 beta (GSK3β) can upregulate DRP1 expression, thereby inducing mitochondrial defects and synaptic injury (87). Consistent with this, disrupting mitochondrial fission can prevent high-fat diet (HFD)-induced obesity (88). Moreover, the expression of the mitophagy-associated protein, PINK1, is decreased in HFD-fed mice, while PINK1 overexpression can improve glucose uptake and downregulate the levels of gluconeogenic enzymes (89). Mice lacking FUNDC1 also develop more severe obesity and insulin insensitivity (90). However, one study proposed the opposite conclusion, namely, that the levels of FUNDC1, IP3R2, and MAM formation were all significantly increased in cardiac tissues from both diabetic patients and diabetic mice. Moreover, *Fundc1* deletion could ameliorate diabetes-induced MAM formation, as well as mitochondrial fragmentation and function (91). That the MAM has an essential role in regulating mitochondrial dynamics and mitophagy suggests that MAM disruption may lead to insulin resistance by inducing mitochondrial dysfunction.

### ER Stress

The ER is the primary site for protein synthesis, folding, processing, and quality control. An imbalance between protein folding requirements and the protein folding capacity of the ER due to physiological demands or disease states will lead to ER stress. To restore proteostasis, cells activate a prosurvival response called the unfolded protein response (UPR). The adaptive UPR activates three parallel signaling branches: the protein kinase R-like ER kinase (PERK) –eukaryotic translation initiation factor 2 alpha (eIF2α) pathway; the IRE1α–X-box binding protein 1 (XBP1) pathway; and the activating transcription factor 6 alpha (ATF6α) pathway. Under resting conditions, the ER-luminal domains of PERK, IRE1α, and ATF6α bind to binding immunoglobulin protein (BiP) and are sequestered as inactive forms. Under stress, however, unfolded proteins bind to BiP, leading to the release of PERK, IRE1α, and ATF6α from BiP, which triggers the UPR (24). The UPR signal limits the protein folding load on the ER, allowing the ER to clear misfolded proteins by transcriptionally expanding its protein folding capacity. However, when the capacity of the UPR is overwhelmed, cells will shift from survival to cell death mode.

The MAM has been implicated in ER stress and the UPR. First, disrupting ER–mitochondria communication can activate the UPR (26). Second, some of the proteins enriched or localized on the MAM have roles in the UPR. For instance, MFN2 can physically interact with PERK, and is an upstream modulator of PERK activity (92). Moreover, VAPB can directly interact with ATF6, thereby attenuating its activity (93). Meanwhile, the P56S mutation in VAPB can disrupt the IRE1α/XBP1 pathway (94). Recently, the newly characterized microprotein PIGBOS, which is localized at ER–mitochondria contact sites, was reported to also regulate the UPR (95). Third, some UPR-associated proteins are localized and function on the MAM. For example, PERK is enriched at the MAM, promoting ER–mitochondria contacts as well as ROS-triggered, mitochondria-mediated apoptosis (96). A fraction of IRE1 can be stabilized and activated by Sig-1R at the MAM under ER stress (97), and the presence of IRE1α on the MAM can determine IP3R availability (93). Furthermore, Sig-1R can also associate with BiP on the MAM, while increased Sig-1R levels can ameliorate ER stress (28). These results suggest that the MAM constitutes an important platform for UPR signaling and plays an essential role in regulating ER stress.

**Figure 5:**
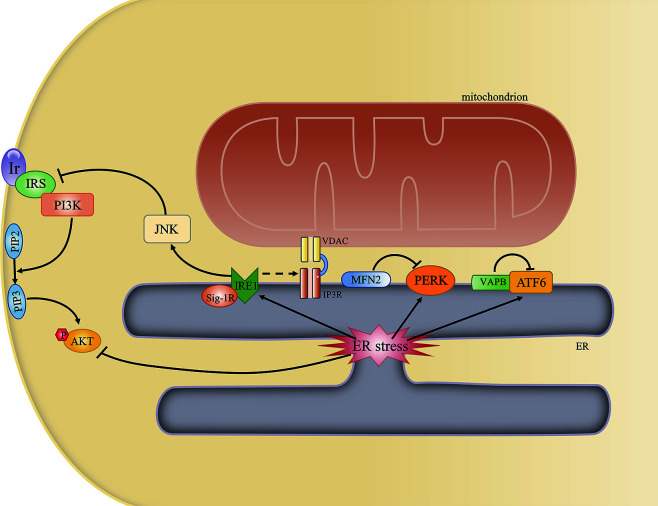
The mitochondria-associated endoplasmic reticulum (ER) membrane (MAM) regulates ER stress and insulin signaling. Some ER stress-associated proteins may localize to the MAM and may be regulated by MAM-resident proteins. An overwhelmed unfolded protein response (UPR) can directly or indirectly inhibit AKT phosphorylation by activating the JNK pathway.

ER stress is a common feature in obesity and diabetes, both of which are characterized by insulin resistance. The phosphorylation of PERK and eIF2α, an indicator of the presence of ER stress, was reported to be significantly increased in HFD-fed and *ob/ob* mice (98). The expression levels of several ER stress-related proteins, as well as that of XBP1 mRNA, were reported to be upregulated in the adipose tissue of obese individuals (99). In addition, the expression of the UPR effectors PERK, IRE1α, and ATF6 is higher in endothelial cells of obese adults when compared with that of nonobese adults (100). Various studies have investigated the cellular mechanisms that link ER stress to insulin resistance. One study proposed that c-Jun N-terminal kinase (JNK) is hyperactivated in an IRE1α-dependent manner under ER stress. Hyperactivated JNK would then promote the serine phosphorylation of IRS-1 and the subsequent reduction in insulin receptor signaling. Meanwhile, AKT phosphorylation is also suppressed under ER stress (98) (**Figure 5**). Another study reported that ER stress also mediates insulin resistance by impairing glucose uptake (101).

Given that the MAM is a key regulator of ER stress and ER stress is one of the main contributors to insulin resistance, it is likely that ER stress represents another mechanism through which MAM impairment can lead to insulin resistance.

### Inflammation

Chronic, low-grade inflammation is a primary cause of insulin resistance. Hypertrophied adipocytes and infiltrated immune cells both contribute to increased blood concentrations of proinflammatory mediators, such as tumor necrosis factor alpha (TNF-α), interleukin 6 (IL-6), IL-1β, leptin, resistin, monocyte chemoattractant protein-1, plasminogen activator inhibitor-1, visfatin, and adiponectin in obese subjects (24). These proinflammatory mediators can directly or indirectly affect inflammation-related pathways, such as the JNK and IKKβ/NF-κB pathways, and also disrupt insulin signaling, eventually leading to systemic insulin resistance and the subsequent development of T2DM (102).

The role of the MAM in inflammation-induced insulin resistance is associated with its role in NLRP3 (nucleotide-binding domain, leucine-rich-repeat-containing family, pyrin domain-containing 3) inflammasome assembly and activation. Activated NLRP3, a pattern recognition receptor, recruits adapter apoptosis-associated speck-like protein containing a caspase-activation recruitment domain (ASC) and subsequently activates caspase-1. Caspase-1 catalyzes the proteolytic activation of IL-1β and IL-18 into active cytokines and initiates pyroptosis (103). The activity of both caspase-1 and IL-1β is increased in adipose tissue of obese animals (104). Furthermore, hepatocytes treated with IL-1β show impaired insulin-induced AKT activation (105).

**Figure 6:**
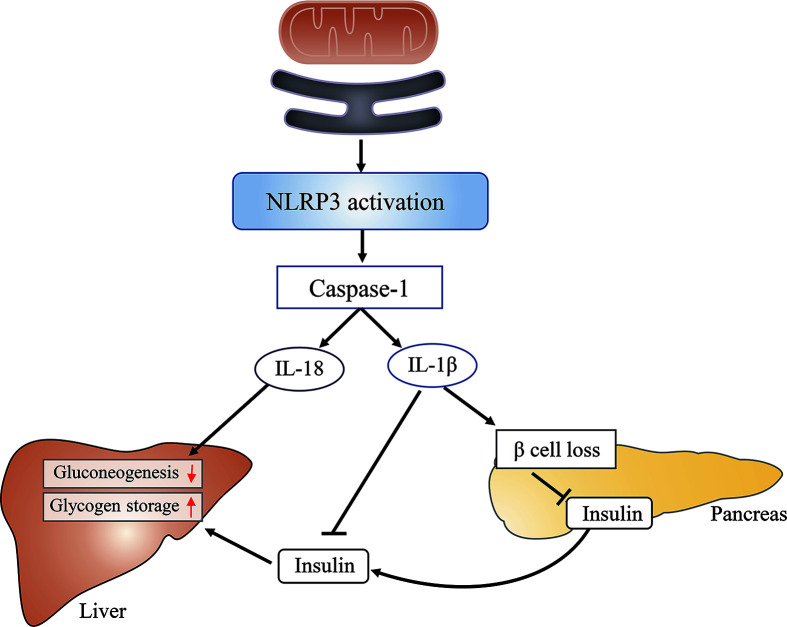
The mitochondria-associated endoplasmic reticulum (ER) membrane (MAM) impairs glucose metabolism by activating the NLRP3 inflammasome. The MAM is required for the activation of the NLRP3 inflammasome, which subsequently activates caspase-1. Caspase-1 catalyzes the proteolytic activation of IL-1β and IL-18. The latter can inhibit hepatic gluconeogenesis, whereas the former induces β-cell loss, reduces insulin secretion, and suppresses insulin action in the liver, finally leading to increased glucose release from hepatocytes and elevated blood glucose levels.

Under normal conditions, most NLRP3 localizes to the ER; however, following inflammatory stimulation with alum or nigericin, NLRP3 and ASC were shown to relocate to the perinuclear space and colocalize with the MAM (106). The possible reasons for the observed NLRP3 relocation to the MAM include that DNA released from the mitochondria (mtDNA) constituted a damage-associated molecular pattern (DAMP) for NLRP3 activation (107) or that ROS were necessary for NLPR3 inflammasome activation (108). Considering that mitochondria constitute the main source of cellular ROS, and that ROS are short-lived and can only act as signaling messengers at short distances (109), NLRP3 should ideally be localized close to mitochondria. Furthermore, knockdown of VDAC can significantly impair NLRP3 inflammasome activation (106) (**Figure 6**). Combined, these results suggest that the MAM is an important regulator of various cellular processes, and MAM dysfunction can lead to insulin resistance through diverse pathways.

## Altered MAM Integrity Contributes to Insulin Resistance in Different Tissues

Besides the shared cellular processes, different tissues have specific metabolic characteristics that determine the extent to which they are affected in T2DM. For instance, proinflammatory cytokines secreted by adipocytes and adipose tissue-resident immune cells are the key mediators in triggering insulin resistance. Impaired insulin signaling leads to increased glycogenolysis and gluconeogenesis in the liver, whereas in skeletal muscle and the brain, it leads to reduced glucose uptake. All these effects may eventually result in elevated blood glucose levels and the development of T2DM. The MAM plays an important role in inducing or aggravating insulin resistance in these tissues.

### Altered MAM Integrity Induces Insulin Resistance in Peripheral Tissues

The liver is one of the main insulin-responsive organs. Insulin induces hepatic glucose oxidation, promotes glycogen storage, and inhibits gluconeogenesis, thereby maintaining the blood glucose level within an appropriate range. Elevated blood glucose levels due to hepatic insulin resistance and the subsequent increase in glucose release are the main contributing factors to hyperglycemia (110). It was recently reported that the MAM participates in the hepatic glucose-sensing system, regulating mitochondrial function during nutritional transition. Chronic disruption of the MAM may contribute to the hepatic mitochondrial dysfunction associated with insulin resistance (66). This is consistent with the results of a previous study showing that cyclophilin D (CYPD, a newly identified partner of the IP3R complex) knockout mice displayed disrupted Ca^2+^ signaling in the liver as well as hepatic insulin resistance. The authors suggested that disrupted Ca^2+^ signaling between the ER and mitochondria linked MAM disruption to hepatic insulin resistance (111). In addition, forced expression of MFN2 ameliorated palmitic acid-induced hepatic insulin resistance, which led to increased ER–mitochondria contacts (112). However, this perspective was challenged by studies demonstrating that enhanced ER–mitochondria interactions are detrimental to insulin signaling. Increased MAM formation in obese animals resulted in mitochondrial Ca^2+^ overload, impaired mitochondrial respiration, and increased oxidative stress. Furthermore, the silencing of the ER–mitochondria tethering proteins PACS2 or IP3R1 improved glucose metabolism (113). Mice lacking PEMT, a MAM-localized PC synthesizing protein, were protected against HFD-induced obesity and insulin resistance (114). However, as PEMT is a functional and not a tethering protein, it may not have been the damaged MAM structure that promoted insulin resistance in this study, but rather the impaired MAM function. Equally, impaired insulin signaling also affects ER–mitochondria interactions, but here the results are also conflicting. An animal study showed that inhibition of AKT/mTOR signaling led to decreased ER–mitochondria contact in HFD-fed mice (115). In contrast, a greater number of hepatic ER–mitochondria contact sites were observed in mice with either nutritionally (HFD) induced or genetically determined obesity compared with their respective lean controls (113). A similar pattern and similar controversial results were found for muscle tissue.

The skeletal muscle is one of the major sites of insulin-mediated glucose uptake and the primary target for alterations in insulin-resistant states (116). The communication between mitochondria and the ER or sarcoplasmic reticulum has been extensively studied in skeletal muscle (117). Interrupting ER–mitochondria contacts is sufficient for the development of muscle insulin resistance. In skeletal muscle of mice with obesity and T2DM, disrupted ER–mitochondria contacts were shown to be an early event preceding mitochondrial dysfunction and insulin resistance, indicating that the disruption of ER–mitochondria coupling may contribute to muscle insulin resistance. In human myotubes, palmitate-induced insulin resistance was associated with impaired MAM integrity (118). In contrast, extensive proteome profiling of mitochondria from skeletal muscle of patients with T2DM illustrated that the expression levels of most of the MAM-localized proteins were upregulated, including that of the MAM tethering protein MFN2 (119). Similarly, it was recently reported that increased pyruvate dehydrogenase kinase 4 (PDK4) activity could suppress insulin signaling by enhancing MAM formation. *Pdk4^−/−^* mice exhibited reduced MAM formation and were protected against diet-induced insulin resistance in skeletal muscle (120). The authors speculated that, in the early stage, increased ER–mitochondria contacts might be beneficial, promoting fatty acid oxidation and ATP production; however, continued MAM formation could lead to a constant rise in mitochondrial Ca^2+^ levels, ROS production, and mitochondrial dysfunction, eventually resulting in insulin resistance (120).

Given the contradictory results reported, it is still unclear whether it is a decrease or an increase in MAM formation that leads to insulin resistance. Eisner et al. suggested that the different findings may have been due to the different species, cell culture conditions, and timelines utilized for analysis given that MAMs display highly dynamic responses to environmental factors and cellular status (117). Moreover, the relationship between altered MAM integrity and insulin resistance may be reciprocal. MAM-resident proteins regulate insulin signaling and vice versa. Whether altered MAM integrity leads to insulin resistance or whether impaired insulin signaling disrupts MAM functions, and which factor is causal, remains to be determined.

### Altered MAM Integrity in Brain Insulin Resistance

The brain is an organ with a high energy demand, while glucose is the primary substrate for brain energy metabolism. Therefore, efficient glucose uptake and utilization, which are regulated by insulin signaling, are essential for brain functions. Unsurprisingly, brain insulin resistance has been widely linked to various neurodegenerative diseases (121–123). However, several studies have demonstrated that brain glucose uptake occurs in an insulin-independent manner. First, glucose transport across the blood–brain barrier is mediated by GLUT1, which is expressed on endothelial cells in brain microvessels and on glial cells (124, 125). GLUT1 activity is dependent on the large glucose concentration gradient that exists between the brain and plasma, and is not regulated by insulin. Moreover, most neurons in the brain express the insulin-independent glucose transporter GLUT3 (126). Second, the insulin-dependent glucose transporters on neurons, such as GLUT4 and GLUT8, are restricted to specific brain regions and some are present at low levels, indicating that insulin-dependent glucose uptake may be brain region-specific (127). Therefore, when mentioning brain insulin resistance, the brain region should be specified.

The hypothalamus is an insulin-sensitive brain region that expresses high levels of insulin receptors and insulin-independent glucose transporters (127, 128). Emerging evidence has shown that the MAM is involved in the development of hypothalamic insulin resistance. Pro-opiomelanocortin (POMC) neurons and agouti-related protein (AgRP) neurons in the hypothalamus can respond to insulin and leptin. JNK activation is thought to be an important mediator of hypothalamic insulin resistance; in turn, hypothalamic insulin resistance contributes to disrupted energy homeostasis as well as obesity (129). The deletion of MFN2 in anorexigenic POMC neurons impaired ER–mitochondria contacts, resulting in defective POMC processing, ER stress-induced leptin resistance, and obesity. Pharmacological relief of hypothalamic ER stress can reverse these metabolic alterations (130). Similarly, Brenda et al. found that, following palmitic acid treatment, ER stress was induced only after MFN2 downregulation (30). These results indicate that ER stress may be the key pathway through which impaired MAM integrity leads to defects in hypothalamic insulin signaling. Conversely, orexigenic AgRP neuron-specific MFN1 or MFN2 knockout mice gained less weight when fed a HFD than their respective controls (131). A recent study demonstrated that, of the 1,313 nonredundant proteins identified in MAMs isolated from the brains of diabetic mice, 144 exhibited significantly altered expression (upregulated or downregulated). These proteins were involved in multiple disease-relevant signaling pathways, such as those associated with the UPR, p53, hypoxia-related transcription factors, and methyl CpG binding protein 2 (132). Furthermore, some UPR-related proteins that can be regulated by the MAM can also affect insulin signaling in the brain. For instance, the constitutive expression of XBP1 in POMC neurons was shown to protect the neurons against diet-induced obesity and also improved insulin sensitivity (133). The loss of IRE1α in POMC neurons accelerates ER stress and predisposes POMC neurons to insulin resistance (134). These results again confirm the central role of ER stress in MAM-induced brain insulin resistance.

Ceramide-induced lipotoxicity is a key ER stress mediator in the hypothalamus. Studies have shown that the levels of ceramides and other lipids known to induce insulin resistance were increased in the hypothalamus of mice fed with a HFD (135). These increased ceramide concentrations led to an increase in the expression of lipotoxicity-related proinflammatory factors. Moreover, this effect could not be reversed by ameliorating ER stress, indicating that the lipotoxicity occurred upstream of ER stress in the hypothalamus (136). Ceramides can also promote hypothalamic insulin resistance *via* activating PKCζ which inhibits AKT phosphorylation (61). These alterations in the hypothalamus can lead to the inhibition of sympathetic activity, resulting in increased hepatic glucose production, decreased brown adipose tissue thermogenesis, and the subsequent weight gain (136, 137). Importantly, most studies investigating the role of the MAM in brain insulin resistance have focused on the hypothalamus, and how the MAM influences insulin signaling in other brain areas remains to be elucidated.

## The MAM as a Potential Therapeutic Target for T2DM Treatment

The fact that the MAM plays a pivotal role in regulating insulin signaling and that alterations in MAM integrity are responsible for insulin resistance, suggests that the MAM represents a potential therapeutic target for the treatment of T2DM. On the one hand, insulin signaling can be improved by reinforcing/downregulating ER–mitochondria contacts. For instance, the overexpression of ER–mitochondria tethering proteins, such as MFN2 and CYPD, can attenuate hepatic insulin resistance (112); in skeletal muscle, meanwhile, the inhibition of PDK4, the activity of which can increase ER–mitochondria contacts, can improve insulin signaling by suppressing MAM formation (120). On the other hand, the increased insulin sensitivity caused by hypoglycemic agents is accompanied by improved ER–mitochondria contacts. Diabetic mice treated with metformin showed increased insulin sensitivity and MAM numbers in the liver (37). Additionally, rosiglitazone treatment can restore VDAC1, CYPD, and PACS2 expression in mice with diet-induced diabetes. However, relatively few studies have investigated whether improved MAM integrity can ameliorate brain insulin resistance and whether the MAM is the target of antidiabetic treatment in the brain. Further research is needed to answer these questions.

Given the diversity of proteins in the MAM and its essential role in regulating insulin signaling, the MAM may be a promising therapeutic target for T2DM treatment.

## Discussion

The MAM, a scaffold between mitochondria and the ER, regulates ER function (such as the UPR), mitochondrial physiology, and metabolite exchange between these organelles. This renders the MAM an important contributor to the maintenance of cellular homeostasis. Altered MAM integrity may lead to insulin resistance through the induction of ER stress, mitochondrial dysfunction, and impaired metabolite synthesis and transport. Current evidence suggests that the MAM is closely associated with insulin resistance in the liver and muscle. Moreover, ER stress may be the key mechanism underlying MAM-induced brain insulin resistance, especially in the hypothalamus. It is also noteworthy that improved MAM integrity may lead to increased insulin sensitivity, while hypoglycemic treatment is associated with improved ER–mitochondria contacts, which indicates that MAM has potential a therapeutic target for T2DM treatment. However, the results to date regarding the influence of the MAM on mitochondrial morphology and insulin signaling are contradictory, and several questions remain unanswered. First, the cause-and-effect roles of altered MAM integrity and insulin resistance remain unclear, as does whether it is enhanced or weakened ER–mitochondria contacts that promote insulin resistance. Second, further studies are needed to investigate how the MAM affects insulin resistance in different brain areas.

## Author Contributions

HC and XZ wrote and prepared the original draft. HC, XZ, XG, GH, YL, YW, and GW wrote, reviewed, and edited the manuscript. HC, XZ, and GW were in charge of the visualization. GW and XZ supervised the study. HC, GW, and XZ were the project administrators. GW and XZ acquired the funding. All authors contributed to the article and approved the submitted version.

## Funding

This work was funded by the National Natural Science Foundation of China (81670732, 81970687, 81900726), the National Key R&D Program of China (2016YFC0901204), the Jilin Provincial Science and Technology Department program (2016C020, 2017C019, 201909010006JC), and the 10th Youth Project of the First Hospital of Jilin University (JDYY102019025).

## Conflict of Interest

The authors declare that the research was conducted in the absence of any commercial or financial relationships that could be construed as a potential conflict of interest.
